# Supplementary material for: Amyloid-like aggregating proteins cause lysosomal defects in neurons via gain-of-function toxicity
Source: Life Sci Alliance. 2021 Dec 21;5(3):e202101185. doi: 10.26508/lsa.202101185 (PMC8711852; doi:10.26508/lsa.202101185)
Supplement: Supplementary file 1 [file LSA-2021-01185_TableS1.docx]

| Assay | Figure | Cell type | Transfection/  Transduction | Time point(s) |
| --- | --- | --- | --- | --- |
| β protein aggregation | Fig. 1A-B  Fig. S1A-D | Neurons  Neurons | Transfection  Transduction | DIV 10+1  DIV 10+3, 10+4, 10+6 |
| β protein toxicity | Fig. 1C-D, S5B-C  Fig. S1E | Neurons  Neurons | Transfection  Transduction | DIV 10+1, 10+3  DIV 10+4 to 10+14 |
| Sholl analysis | Fig. 1E-F | Neurons | Transfection | DIV 10+2 |
| Cryo-ET | Fig. 2, 3, S3, S4  Fig. S3 | Neurons  HeLa | Transfection  Transfection | DIV 6+1  24 hours after transfection |
| Lysotracker | Fig. 4  Fig. 8E-F | Neurons  Mocha cells | Transfection  None | DIV 6+1  Day 1 after plating |
| LC3 +/- chloroquine | Fig. 5A-C | HeLa | Transfection | 48 hours after transfection |
| mCherry-GFP-LC3 reporter | Fig. 5D-G | Neurons | Transfection | DIV 10+3 |
| Western blot, RT-PCR for autophagy markers | Fig. 5H-J | HeLa | Transfection | 24 hours after transfection |
| β protein interactome and total proteome | Fig. 6A-H, S6, S7A-D, F | Neurons | Transduction | DIV 10+4 |
| AP-3μ1 / β protein colocalization | Fig. 7A-B | Neurons | Transfection | DIV 10+2 |
| Western blots for AP-3μ1 in soluble and pellet fraction | Fig. 7C-E | Neurons | Transduction | DIV 10+6 |
| TauRD seeding | Fig. 7F-J | Neurons | Transduction | DIV 10+7 |
| Western blot for other AP-3 subunits | Fig. 8A-B | Neurons | Transduction | DIV 10+6 |
| β protein / AP-3μ1 co-IP | Fig. S7E | Neurons | Transduction | DIV 10+7 |
| Lamp1 localization | Fig. 8C-D, S7G | Neurons | Transduction | DIV 10+4, 10+5 |
| AP-3 subunits in mocha cells | Fig. S8A-C | Mocha cells | None | Day 1 after plating |
| Toxicity rescue by AP-3μ1 | Fig. S8D-E | Neurons | Transfection | DIV 6+1 |
