# Supplementary material for: Amyloid-like aggregating proteins cause lysosomal defects in neurons via gain-of-function toxicity
Source: Life Sci Alliance. 2021 Dec 21;5(3):e202101185. doi: 10.26508/lsa.202101185 (PMC8711852; doi:10.26508/lsa.202101185)
Supplement: Supplementary file 2 [file LSA-2021-01185_TableS2.docx]

| Data analyzed | Cell type | Condition | Number of experiments | Number of cells | Number of observations |
| --- | --- | --- | --- | --- | --- |
| Aggregates | Neurons  Neurons  HeLa | β4-mCherry  β23-mCherry  β23-mCherry | 3  2  4 | 9  2  7 | 21  4  23 |
| Lysosomes analyzed for aggregate content | Neurons  Neurons | β4-mCherry  β23-mCherry | 5  3 | 10  5 | 38  31 |
| Early autophagosomes | Neurons  Neurons  Neurons | mCherry  β4-mCherry  β23-mCherry | 5  5  4 | 14  15  9 | 1  2  0 |
